# Ascites in Children

**Published:** 1887-09

**Authors:** 


### *ASCITES IN CHILDREN.*

Ascites in children is a rare disease. If it happens, it is generally produced either by tubercular peritonitis, or by cancer of some of the abdominal organs. Dr. Seiler, in Dresden, contends *Berl. Klin. Wochenschr.*, xviii., 26, p. 365) that whenever the causes just mentioned cannot be detected in ascites of children, a diffused syphilitic hepatitis or circumscribed gummata will *always* be found to have given rise to the abdominal dropsy. He thinks that the cases so far reported belong to the category of retarded hereditary syphilis, and that this form of ascites always yields to mercury or iodine, or to a combination of both. At the same time, he admits that occasionally this ascites may be caused by a curable, simple hypertrophic cirrhosis of the liver. For better explanation, Seiler gives the history of four cases:

1. A girl, aged 13, after having frequently complained of not feeling well, without the appearance of any specific signs or symptoms, was taken sick, February of last year, with swelling of the abdomen, which on examination was found to be distended with a rather large quantity of free fluid. June 11th, about five quarts of a serous, light yellow fluid, containing a considerable percentage of albumen, was withdrawn, by paracentesis, from the abdominal cavity, after which operation the liver could be felt as a soft tumor extending down below as far as the middle of the abdomen. Mercurial inunctions into the abdominal walls, and internal administration of iodide of potash, caused a diminution in the size of the liver, and the ascites did not reappear. Cure within six weeks.

2. A four-year-old girl, with really enormous ascites; otherwise perfectly healthy. By paracentesis, four quarts of turbid serum was withdrawn, when it was found that the liver reached as far as the crista ilei. Seven weeks later, tapping had again to be resorted to, but this time the quantity of the fluid was less. The same treatment as in the first case was continued for two months, when a tonic treatment was instituted. When, eight months after its admission, the child was discharged from the hospital, the ascites had disappeared, but the liver was still a little lower down than the middle of the abdomen. Seiler saw the girl again when she was thirteen years old, and, on percussion, found the size of the liver to be normal. The ascites had never returned.

The third and fourth cases concern a seven and a fifteen-year-old girl, and contain nothing of special interest, their history being similar to the first two cases.

That there may be still other causes producing ascites in children, there can be no doubt; the writer of this saw one in which, in consequence of intense inflammation of the portal vein (due to a cold?) and occlusion of the same near its entrance into the liver, the most intense ascites developed itself in a boy nine years old. The case ended fatally. Kormann has also reported another case (*Jahrb. f. Khkde.*, xvi., p. 170, 1880,) in which the etiology did not coincide with that given by Seiler.—*Medical and Surgical Reporter.*

